# Long-Term Beetroot Extract Supplementation Improves Morphological Muscle Quality and Rate of Force Development in Postmenopausal Women: A Randomized Clinical Trial

**DOI:** 10.3390/nu18050860

**Published:** 2026-03-07

**Authors:** Olavo João Frederico Ramos Junior, Carlos Alberto de Souza Filho, Shaheen Majeed, Thiago Silveira Alvares

**Affiliations:** 1 Nutrition and Exercise Metabolism Research Group, Multidisciplinary Center UFRJ-Macaé, Federal University of Rio de Janeiro, Macaé 21941-901, Rio de Janeiro, Brazil; 2 Multicenter Graduate Program in Physiological Sciences, Federal University of Rio de Janeiro, Macaé 21941-901, Rio de Janeiro, Brazil; 3 Sabinsa Corporation, 20 Lake Drive, East Windsor, NJ 08520, USA; 4 Food and Nutrition Institute, Multidisciplinary Center UFRJ-Macaé, Federal University of Rio de Janeiro, Macaé 21941-901, Rio de Janeiro, Brazil

**Keywords:** functional foods, dietary supplements, vegetables, muscle function, nitric oxide, aging

## Abstract

**Background:** Low estrogen levels during menopause reduce nitric oxide (NO) production, contributing to decline in skeletal muscle quality and function. Although acute and short-term dietary nitrate supplementation has demonstrated promising effects, long-term benefits, particularly on muscle quality in postmenopausal women, are not well established. **Objectives:** The objective was to investigate the effects of long-term (12-week) nitrate-rich beetroot extract supplementation on morphological and functional muscle quality, rate of force development (RFD), maximal strength, and circulating nitrate/nitrite concentrations in postmenopausal women. **Methods:** In a randomized, double-blind, placebo-controlled design, 20 postmenopausal women (21 years ± 7 since menopause) consumed 20 g/day of a nitrate-rich beetroot extract (BET; 548 mg nitrate/day) or a nitrate-depleted beetroot extract (PLA; 43 mg nitrate/day) for 12 weeks. Outcome measures, including muscle quality (functional via muscle strength/thickness ratio; morphological via ultrasound echo intensity), RFD, maximal voluntary isometric contraction (MVIC), and serum nitrate/nitrite levels, were evaluated at baseline, 8 weeks, and 12 weeks. **Results:** BET significantly increased serum nitrate (0.005) and nitrite (0.022) levels compared to PLA at both week 8 and week 12. Morphological muscle quality also improved significantly in the BET group (interaction effect, *p* = 0.014). Early-phase rate of force development (RFD) increased between 30 and 100 ms, whereas late-phase RFD increased between 100 and 200 ms. RFD_peak_ also improved by week 8, and these gains were maintained through week 12 (interaction effect, *p* < 0.05). Although there was no significant difference between groups for functional muscle quality, MVIC increased at week 12 in the BET group, but no significant Time × Group interaction was observed. **Conclusions:** Twelve weeks of nitrate-rich beetroot extract supplementation improved morphological muscle quality and RFD, suggesting potential clinical relevance for preventing structural and neuromuscular function decline in postmenopausal women. This study was registered with ReBEC (RBR-87qh649) and approved on 8 October 2024.

## 1. Introduction

Aging induces progressive alterations in skeletal muscle characterized by reductions in muscle quality and strength, which can ultimately culminate in sarcopenia, a syndrome linked to increased frailty and impaired physical function in older adults [1]. These age-related muscular changes encompass both functional deficits and structural remodeling, including an increase in intramuscular fat and fibrous tissue, alongside the loss of muscle mass, all of which may contribute to reduced muscle quality [2]. Postmenopausal females, particularly, experience more pronounced muscle impairments largely due to the steep decline in estrogen levels that influence muscle maintenance mechanisms [3]. Estrogen contributes to skeletal muscle health by facilitating protein synthesis, preserving satellite cell function, attenuating catabolic pathways, and regulating inflammation, processes that collectively support muscle integrity and function [4].

Declining muscle quality is strongly implicated in sarcopenia, which manifests clinically as decreased physical capacity, impaired postural balance, reduced ability to perform daily tasks, and increased risk of falls [5]. Additionally, neuromuscular decline during aging may lead to a reduction in the rate of force development (RFD), which reflects the capacity of the neuromuscular system to rapidly generate force [6]. This decline in RFD has been correlated with increased fall susceptibility and reduced mobility among older females, underscoring its relevance for maintaining functional independence [7,8,9]. Therefore, interventions that target improvements in muscle quality and neuromuscular performance are important to mitigate age-related functional decline.

Dietary nitrate, present in vegetables such as beetroot and spinach, represents a promising nutritional approach to improve nitric oxide (NO) bioavailability via the nitrate-nitrite-NO pathway, thereby improving muscle perfusion, oxygen delivery, and contractile efficiency [10]. Acute and short-term beetroot juice intake has demonstrated benefits in endothelial function and muscle contractility in older populations [11,12,13]. Furthermore, 8-week interventions have shown that nitrate-rich beetroot extract elevates fasting plasma nitrate and nitrite levels, accompanied by improvements in muscle strength and power in postmenopausal women [14]. Recent systematic reviews and meta-analyses support the neuromuscular benefits of dietary nitrate supplementation, particularly for muscle power and contractile function; however, they also highlight a lack of long-term randomized trials focusing specifically on postmenopausal women and clinically relevant muscle quality outcomes [15,16]

NO plays a critical role in skeletal muscle performance by modulating Ca^2+^ release and sensitivity, thereby enhancing muscle contractile efficiency and functional capacity [17]. However, aging, particularly when accompanied by a reduction in estrogen, is associated with decreased expression of NO synthase 1 (NOS1) in muscle, reduced NO bioavailability, and lower concentrations of its metabolic precursors (nitrate and nitrite) [18]. These physiological alterations may contribute to muscle weakness, reduced exercise capacity, and an increased risk of sarcopenia [2,18].

Despite established acute and short-term advantages of beetroot consumption on NO bioavailability and muscle function, there remains limited evidence regarding the long-term effects of nitrate supplementation on both morphological and functional muscle quality, as well as rate of force development, in postmenopausal women.

Therefore, the present study aimed to investigate whether 12 weeks of nitrate-rich beetroot extract supplementation could improve functional and morphological aspects of muscle quality, the rate of force development, and maximal strength in postmenopausal older women. We hypothesized that prolonged supplementation with beetroot extract would lead to sustained improvements in muscle quality and strength outcomes in this population.

## 2. Methods

### 2.1. Participants

Participant characteristics and detailed methodological procedures for this cohort have been described previously in a companion manuscript investigating the effects of dietary nitrate supplementation on skeletal muscle contractile properties in postmenopausal women [14]. The present study expands on this work by examining additional outcomes related to muscle quality and rate of force development. Twenty-five postmenopausal women (21 years ± 7 since menopause) were recruited to participate in the study. However, 5 participants dropped out across the visits; thus, data from twenty participants are reported. Eligibility criteria included older females (60 to 85 years old) who were postmenopausal for at least one year, free from hormone replacement therapy, non-smokers, and without contraindications to exercise. The study protocol complied with the principles of the Declaration of Helsinki and was approved by the Institutional Ethics Committee of the Federal University of Rio de Janeiro (CAAE 55245622.2.0000.5699) on 18 March 2024. The trial was registered in the Brazilian Registry of Clinical Trials (ReBEC; RBR-87qh649; https://ensaiosclinicos.gov.br/rg/RBR-87qh649 (accessed on 2 February 2026)) and approved on 8 October 2024. The study was prospectively registered, and participant recruitment began only after trial registration approval (October 2024) and continued for 12 months. All participants provided written informed consent prior to participation.

### 2.2. Study Design

**Figure 1:**
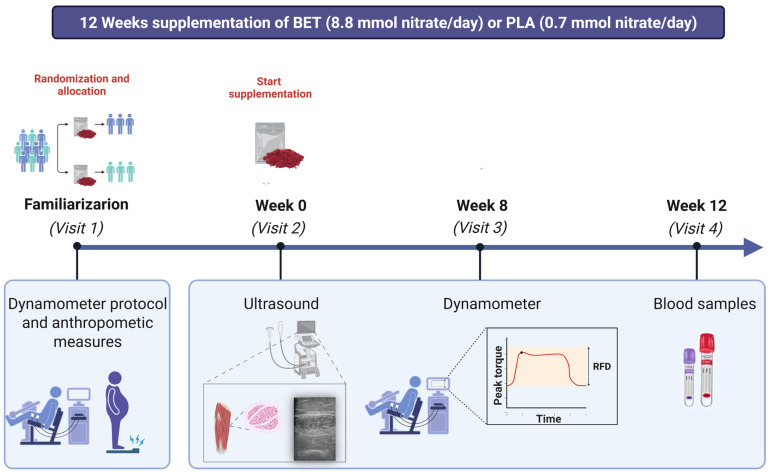
Schematic illustration of study design.

Participants were assigned to receive either a nitrate-rich standardized beetroot extract (BET) or a nitrate-depleted beetroot extract (PLA) in a randomized, double-blind, placebo-controlled, and parallel-group manner (Figure 1). A total of 25 participants were assessed for eligibility and randomized (PLA: n = 12; BET: n = 13). Five participants discontinued the intervention (PLA: n = 2; BET: n = 3) due to upper respiratory tract infection (n = 1), lower limb injury (n = 1), and non-adherence to supplementation (n = 3). Therefore, 20 participants (n = 10 per group) completed the study and were included in the final analysis. Baseline physical activity was assessed using the International Physical Activity Questionnaire (IPAQ). Participants were instructed to maintain their usual activity levels throughout the study. Baseline assessments included venous blood sampling for serum nitrate and nitrite levels, ultrasound evaluation of muscle architecture, and isokinetic dynamometry to measure muscle function. Participants began supplementation with a nitrate-rich beetroot extract (Sabeet^®^, Sabinsa Corporation, East Windsor, NJ, USA) immediately after the baseline measurement, consuming 10 g of the assigned extract dissolved in 250 mL of water twice daily for 12 weeks. The nitrate-rich beetroot extract contained approximately 548 mg (8.8 mmol) of nitrate per day, while the nitrate-depleted beetroot extract contained about 43 mg (0.7 mmol) of nitrate daily. Supplements were dispensed weekly, and adherence, as well as adverse events, were monitored. Participants received their supplement supply each week and returned any unused sachets at the following visit. Compliance was calculated based on the number of sachets consumed. Follow-up evaluations were conducted at weeks 8 and 12 post-baseline. One day prior to the beginning of the study, participants received detailed instructions on dietary nitrate and nitrite content, including foods to avoid and foods recommended for a low nitrate/nitrite diet throughout the intervention. A comprehensive list, derived from established databases of nitrate and nitrite content in animal- and plant-based foods, was provided to facilitate dietary compliance and minimize confounding dietary nitrate intake [19]. Dietary intake was monitored using repeated 24-h dietary recalls at baseline, week 8, and week 12. Detailed data on dietary nitrate intake, nitrate provided by the intervention, and total daily nitrate intake are presented in Supplementary Table S3. To maintain nitrate conversion efficiency, participants avoided tongue scraping, chewing gum, and antibacterial mouthwash throughout the study and were instructed to continue their normal daily activities. Before each assessment, participants fasted for 12 h and abstained from physical exercise for 24 h. Participants, outcome assessors, and data analysts were blinded to group allocation. Supplements were provided in identical sachets, and the randomization code was held by an independent researcher until study completion. Ultrasound images were analyzed by an investigator blinded to group and time point.

### 2.3. Muscle Strength and Rate of Force Development Assessment

Maximal voluntary isometric contraction (MVIC) of the dominant knee extensors was measured using an isokinetic dynamometer (Humac Norm, CSMi Medical Solutions, Stoughton, MA, USA). The dynamometer was individually adjusted for each participant, and all settings were carefully recorded to ensure consistent positioning across subsequent testing sessions. Participants performed four maximal isometric contractions at 70° knee flexion with a 30-s rest interval. The highest peak torque was recorded and normalized to body weight. Rate of force development (RFD) was computed from the torque-time curve slopes over time intervals of 0–30, 0–50, 0–100, and 0–200 milliseconds, as well as peak RFD during contractions [20]. The average across repetitions was used for analysis.

### 2.4. Ultrasound Assessment

Muscle thickness. Changes in the non-dominant knee extensor muscle thickness (MT) in each visit were measured using B-mode ultrasonography (LOGIQe, GE HealthCare, Chicago, IL, USA) with a 5.0–10.0 MHz linear-array probe [21]. Participants rested in the supine position for 10 min before images were acquired. The ultrasound probe was coated with a water-soluble gel and positioned transversely and perpendicularly over the measurement site on the anterior surface of the leg, where the muscle thickness (MT) of the vastus intermedius (VI), rectus femoris (RF), vastus lateralis (VL), and vastus medialis (VM) was recorded. The measuring location for the knee extensor muscles was identified as the midpoint between the anterior superior iliac spine and the middle of the knee [22]. The muscle tissue interface between the bone and adipose tissue was identified, and the image on the monitor was frozen. With the image frozen, a cursor was enabled to measure MT. Three MT measures were taken and averaged to obtain a final MT value for each muscle. The overall quadriceps muscle thickness was calculated following the equation $\frac{RF+VI+VL+VM\;}{4}$ and included in the statistical analysis. For consistency, the same person performed the ultrasound image and measuring point identification in all testing visits. Corrected EI was calculated as uncorrected EI + (subcutaneous fat thickness [cm] × 40.5278) [21,23].

Functional muscle quality. Muscle quality is a more sensitive marker of muscle function than muscle strength or muscle mass alone [24]. The functional domain of muscle quality is defined by the muscle function delivered per unit of muscle mass, such as the ratio between maximal muscle strength and total, appendicular, or specific muscle mass [25]. MQ_f_ were calculated by dividing the MVIC of each participant (as described above in the maximal muscular strength assessment section) by all the knee extensor muscle thickness [21], as follows $\frac{MVIC}{MT}$.

Morphological muscle quality. MQ_m_ refers to micro- and macroscopic changes in muscle architecture and composition and was assessed on an ultrasound device using post hoc analysis of density by quantifying echo intensity (EI) [25]. The same image recorded for MT measurement was used to assay EI, with image depth (4.0 cm) and gain (60 dB) settings kept the same for all measures. Transverse ultrasound images were obtained from the anterior region of the knee extensors with participants in a standardized supine position and muscles at rest, following previously described procedures. For EI analysis, a region of interest (ROI) was manually selected within the muscle, carefully avoiding visible fascia and bone. The mean EI was quantified using a gray-scale analysis function and expressed in arbitrary units (a.u.) as a value between 0 (black) and 255 (white) using ImageJ software (National Institute of Health, Bethesda, MD, USA, Version 1.37) [21]. The increase in EI (i.e., a brighter image) in response to exercise has been suggested to represent muscle damage and inflammation, whereas a lower EI indicates a greater density of contractile units within the muscle [26].

### 2.5. Blood Sampling, Nitrate and Nitrite Assessment

Venous blood samples were collected after overnight fasting at baseline, week 8, and week 12, then immediately centrifuged, serum was separated, deproteinized, and stored at −80 °C until analysis. Serum nitrate and nitrite concentrations were quantified using a high-performance liquid chromatography (HPLC) system, which involved protein precipitation, chemical derivatization, and detection by photodiode array for nitrate and fluorescence for nitrite [14].

### 2.6. Statistical Analysis

An a priori power analysis was conducted using G*Power software (version 3.1) for a repeated-measures ANOVA (within–between interaction). Statistical power was set at 1 − β = 0.80, with an effect size of f = 0.39, based on our previous studies in postmenopausal women [14], and a significance level of α = 0.05. The analysis indicated that a minimum sample size of 14 participants was required to avoid a type 2 statistical error. A two-way repeated-measures ANOVA was conducted to evaluate differences in muscle quality, maximal voluntary isometric contraction, rate of force development, and plasma nitrate and nitrite levels between the BET and PLA groups across three time points: baseline (week 0), week 8, and week 12. For rate of force development, each RFD interval was treated as a predefined distinct endpoint, and multiplicity was controlled within each model using the Holm–Šidák adjustment. When a significant F was found, additional post hoc tests with Holm–Sidak’s multiple comparisons test adjustment were performed. Statistical significance was set at the 0.05 level of confidence. All analyses were performed using a commercially available statistical package (IBM SPSS Statistics version 26 for Mac). The graphics were designed using GraphPad Prism 9. The results were expressed as means ± SD.

## 3. Results

Baseline characteristics, including body composition and physical activity levels, were similar between the nitrate-rich beetroot extract group and the placebo group (Table 1).

### 3.1. Muscular Strength Assessment

**Figure 2:**
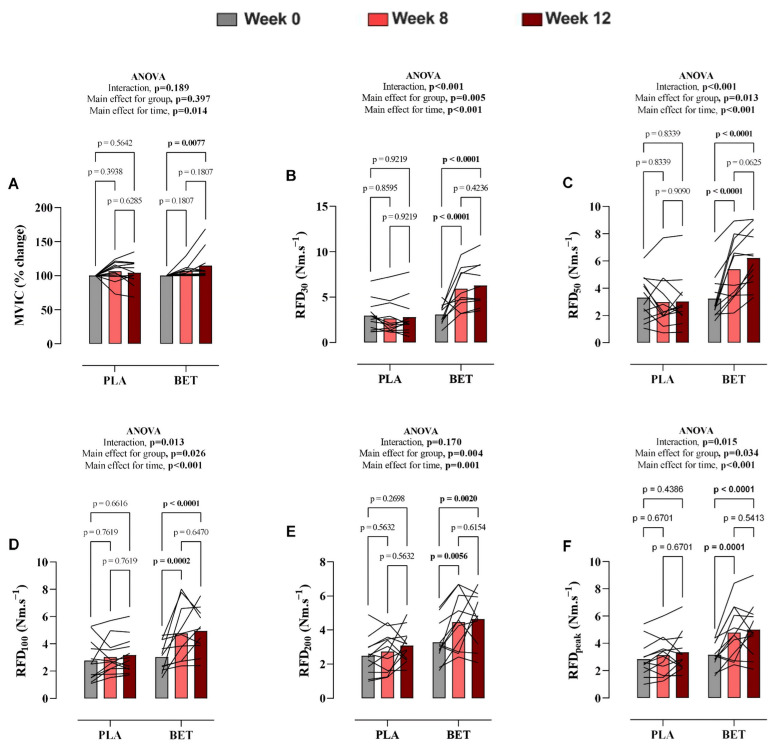
Changes in (**A**) maximal voluntary isometric contraction (MVIC), (**B**) rate of force development (RFD) in the first 0–30 milliseconds (ms), (**C**) RFD in the first 0–50 ms, (**D**) RFD in the first 0–100 ms, (**E**) RFD in the first 0–200 ms, and (**F**) peak RFD levels over the twelve-week study period in the nitrate-rich beetroot extract and nitrate-depleted beetroot extract (placebo) groups. Data presented as mean ± SD. A repeated-measures two-way ANOVA was used to identify differences between the beetroot extract (BET) and placebo (PLA) groups.

Maximal voluntary isometric contraction. Changes in MVIC over the 12-week intervention are presented in Supplementary Table S1 and Figure 2. Although there was no significant time vs. treatment effect for MVIC (*p* = 0.189, η^2^p = 0.08), there was a significant main effect of time (*p* = 0.015, η^2^p = 0.18), with the BET group exhibiting an increase in MVIC at week 12 compared to baseline. No main effect of group was observed (*p* = 0.397). The overall mean difference between groups was −3.66% (95% CI: −12.48 to 5.15).

**Figure 3:**
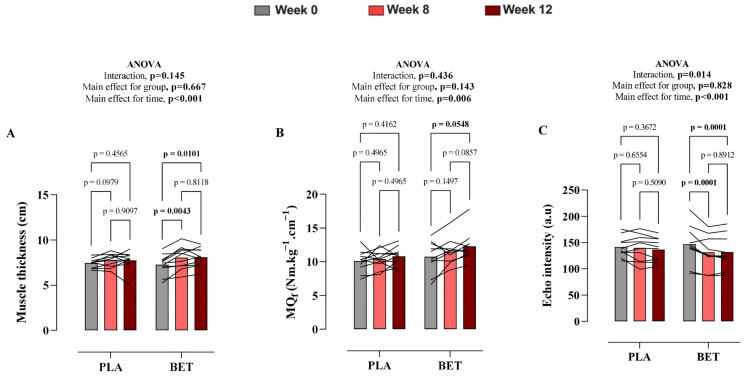
Changes in (**A**) Muscle thickness (MT), (**B**) Functional muscle quality (MQ_f_), and (**C**) Morphological muscle quality (MQ_m_) over the twelve-week study period in nitrate-rich beetroot extract and nitrate-depleted beetroot extract (placebo) groups. Data presented as mean ± SD. A repeated-measures two-way ANOVA was used to identify differences between the beetroot extract (BET) and placebo (PLA) groups.

Rate of Force Development. Rate of Force Development. Data on absolute RFD values at various time intervals (30, 50, 100, and 200 ms) and peak RFD (RFD_peak_) are summarized in Supplementary Table S1 and Figure 2. There was a significant interaction effect for RFD_30_ (*p* < 0.001, η^2^p = 0.47), RFD_50_ (*p* < 0.001, η^2^p = 0.42), RFD_100_ (*p* = 0.013, η^2^p = 0.18), and RFD_peak_ (*p* = 0.015, η^2^p = 0.17), whereas no significant interaction was observed for RFD_200_ (*p* = 0.170, η^2^p = 0.06). Following beetroot extract supplementation, RFD_30_, RFD_50_, RFD_100_, and RFD_peak_ significantly increased at weeks 8 and 12 (Figure 3). The overall mean differences between groups were −2.30 Nm.s^−1^ (95% CI: −3.84 to −0.76) for RFD_30_, −1.84 (95% CI: −3.27 to −0.42) for RFD_50_, −1.25 (95% CI: −2.35 to −0.16) for RFD_100_, −0.67 (95% CI: −1.56 to 0.22) for RFD_200_, and −1.22 (95% CI: −2.33 to −0.10) for RFD_peak_. In contrast, no significant changes were observed in these parameters in the placebo group.

### 3.2. Ultrasound Assessment

Muscle Thickness. Changes in the muscle thickness between the BET and PLA groups following the intervention period are shown in Supplementary Table S1 and Figure 3. There was no significant time × treatment effect for muscle thickness (*p* = 0.145, η^2^p = 0.09). The overall mean difference between groups was −0.16 cm (95% CI: −0.94 to 0.62). However, there was a significant main effect of time, with the BET group exhibiting an increase in muscle thickness at week 8 and week 12 compared to baseline.

Functional muscle quality. Changes in functional muscle quality following the intervention period are shown in Supplementary Table S1 and Figure 3. There was no significant interaction effect for functional muscle quality (*p* = 0.436, η^2^p = 0.04). The overall mean difference between groups was −1.08 Nm·cm (95% CI: −2.56 to 0.40). However, a significant main effect of time was detected. Post hoc analysis revealed a significant increase in the functional muscle quality at week 12 only in the BET group compared to baseline.

Morphological muscle quality. A significant time × treatment interaction was observed for morphological muscle quality, as assessed by ultrasound echo intensity, as presented in Supplementary Table S1 and Figure 3 (*p* = 0.014, η^2^p = 0.21). The overall mean difference between groups was 2.71 a.u. (95% CI: −23.20 to 28.61). Following beetroot extract supplementation, echo intensity significantly decreased at week 8 and week 12 (Figure 3). There were no significant changes in morphological muscle quality in the placebo group.

### 3.3. Serum Nitrate and Nitrite

**Figure 4:**
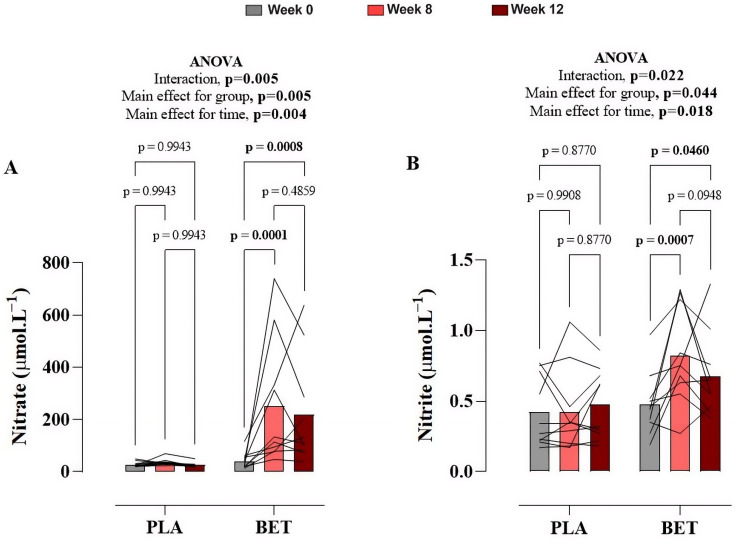
Temporal alterations in (**A**) circulating serum nitrate and (**B**) nitrite concentrations over the twelve-week intervention period in cohorts administered nitrate-rich beetroot extract versus nitrate-depleted beetroot extract (placebo). Data presented as mean ± SD. A repeated-measures two-way ANOVA was used to identify differences between the beetroot extract (BET) and placebo (PLA) groups.

Figure 4 and Supplementary Table S2 describe changes in serum nitrate and nitrite concentrations in the BET and PLA groups over the 12-week study period. There was a significant interaction effect for both serum nitrate (*p* = 0.005, η^2^p = 0.56) and nitrite (*p* = 0.022, η^2^p = 0.37). For serum nitrate, post hoc comparisons demonstrated that overall concentrations were significantly higher in the BET group compared to PLA, with a mean difference of −140.3 μmol L^−1^ (95% CI: −233.9 to −46.76). Similarly, serum nitrite concentrations were significantly elevated in the BET group compared to PLA, with a mean difference of −0.2187 μmol L^−1^ (95% CI: −0.4316 to −0.0058). Post hoc analysis revealed that in the BET group, serum nitrate and nitrite levels were significantly elevated at week 8 and week 12, compared to the PLA group (Figure 4).

## 4. Discussion

The present study investigated the effects of 12-week supplementation with nitrate-rich beetroot extract on morphological muscle quality, rate of force development, maximal strength, and circulating nitrate and nitrite levels in older postmenopausal women. The standardized beetroot extract was well tolerated and well accepted by the participants throughout the intervention period. Our main findings revealed that supplementation with nitrate-rich beetroot extract improves rate of force development and morphological muscle quality, as well as increases serum nitrate and nitrite levels. These findings provide novel evidence supporting the use of prolonged dietary nitrate supplementation as a practical nutritional strategy for mitigating age-related declines in muscle composition and neuromuscular performance.

The observed improvements in morphological muscle quality likely result from multifaceted physiological mechanisms primarily mediated by increased NO bioavailability. NO serves as a crucial regulator of vascular function, promoting vasodilation and enhancing blood flow to skeletal muscles, which are often compromised during aging due to endothelial dysfunction and reduced NO synthase activity [18,27]. Enhanced muscle perfusion ensures adequate nutrient and oxygen delivery, thereby reducing the accumulation of intramuscular adipose tissue and fibrotic infiltration, which negatively impact muscle quality [22,23]. Furthermore, the anti-inflammatory properties of NO may attenuate chronic inflammation associated with aging, minimizing fibrosis and fatty infiltration [18]. At the cellular level, NO influences mitochondrial efficiency and biogenesis through signaling pathways involving peroxisome proliferator-activated receptor gamma coactivator-1 alpha (PGC-1α), which supports mitochondrial health and energy metabolism [4]. Additionally, NO positively regulates anabolic processes, such as muscle protein synthesis via Akt/mTOR signaling pathways, and satellite cell proliferation, critical for muscle repair and regeneration, particularly in estrogen-deficient postmenopausal populations [2,28]. However, it is important to note that echo intensity is an indirect marker of muscle composition, and since gold-standard methods such as MRI or muscle biopsy were not performed, the underlying tissue-level mechanisms should be interpreted with caution. In contrast, functional muscle quality did not show a significant interaction with nitrate-rich beetroot extract supplementation. Although some chronic supplementation studies, particularly those involving amino acids [29] and protein [30], have reported improvements in this variable, these interventions were typically combined with structured resistance training protocols. In the present study, despite the participants being physically active postmenopausal women, no structured resistance training program or progressive load control was implemented. Because functional muscle quality is derived from both muscle thickness and MVIC, and both variables showed a similar pattern over time, it is plausible that, although muscle morphology assessed by echo intensity improved with beetroot extract supplementation, a progressive resistance training stimulus would be necessary in this population to induce meaningful functional adaptations. Therefore, future studies combining nitrate supplementation with structured resistance training are warranted.

Significant improvements were observed in the early-phase rate of force development, suggesting enhanced neuromuscular function, which is important for rapid force production and functional movements such as balance recovery and fall prevention [20]. The early-phase rate of force development (<100 ms) predominantly reflects neural activation, including motor unit recruitment speed and firing rate, both of which are potentially augmented by NO-mediated improvements in neuromuscular transmission [20,31]. Increased NO may enhance calcium sensitivity and release from the sarcoplasmic reticulum through S-nitrosylation of contractile proteins, improving excitation-contraction coupling and force-generating capacity [27,32]. Moreover, an improvement was observed in the peak rate of force development and the later phase (>100 ms), which is more related to structural factors such as muscle architecture and length [18]. This highlights that long-term supplementation with beetroot extract can benefit both neuromuscular function and structure related to exercise.

In that regard, changes in maximal voluntary isometric contraction (MVIC) were observed over time, without a significant Time × Group interaction. Similarly, improvements in muscle thickness and functional muscle quality were driven by a main effect of time across the 12-week intervention, irrespective of group. These findings are consistent with meta-analytical evidence suggesting that dietary nitrate supplementation may be associated with improvements in isometric muscle strength under certain conditions [16,33,34]. Chronic supplementation with nitrate-rich beetroot extract may contribute to functional muscle quality in postmenopausal women by increasing nitric oxide (NO) bioavailability. Enhanced NO availability may improve skeletal muscle perfusion, facilitating oxygen and nutrient delivery, as well as supporting contractile efficiency and mitochondrial function [35]. Moreover, the lack of additional group-related effects on MVIC alongside improvements in explosive strength outcomes may suggest that NO-mediated adaptations preferentially influence mechanisms associated with rapid force production rather than maximal force capacity. This may be partially explained by NO-related modulation of calcium handling and excitation–contraction coupling [36], processes that are particularly relevant for early-phase force development and type II fiber function [37], although these mechanisms remain speculative and warrant further investigation. Collectively, these mechanisms may favor torque and power generation during daily activities and exercise, thereby contributing to the maintenance of muscle quality in this population [14].

The sustained increase in circulating nitrate and nitrite concentrations at week 8 and week 12 corroborates earlier studies demonstrating that chronic dietary nitrate supplementation effectively maintains or enhances NO availability in older populations [14,38]. A randomized controlled trial conducted in postmenopausal women demonstrated a significant elevation in circulating serum nitrate and nitrite concentrations following prolonged supplementation with beetroot extract. These biochemical enhancements were concomitant with a reduction in carotid artery stiffness, indicative of improved vascular function, while systemic blood pressure remained unaltered [38]. Furthermore, recent evidence indicates that an eight-week regimen of nitrate-rich beetroot extract supplementation not only elevated fasting plasma nitrate and nitrite levels but also elicited significant improvements in skeletal muscle contractile properties in this population, thereby highlighting the therapeutic potential of dietary nitrate in ameliorating age-related declines in muscle function [14]. In summary, these findings support the hypothesis that chronic dietary nitrate supplementation enhances nitric oxide–mediated signaling pathways, which are fundamental for preserving vascular perfusion and muscular function in older adults.

Our study has a few limitations. Firstly, the relatively small sample size limits the generalizability and statistical power for detecting smaller differences. Additionally, participants were physically active, which may not represent sedentary or clinically compromised populations. Muscle morphological quality was assessed using ultrasound-derived echo intensity, which, although widely used as a non-invasive indicator of muscle quality, provides an indirect estimate of tissue composition. Therefore, structural muscle findings should be interpreted cautiously. Future studies should include larger and more diverse populations, along with direct assessments of neuromuscular activation, muscle biopsy parameters, and cellular function, to clarify the mechanisms by which dietary nitrate influences muscle health in aging, particularly in postmenopausal women.

## 5. Conclusions

This study demonstrated that 12 weeks of supplementation with nitrate-rich beetroot extract was associated with improvements in morphological muscle quality and rate of force development in older postmenopausal women, particularly during the early phase of RFD. These adaptations are likely mediated, at least in part, by enhanced nitric oxide bioavailability, which may improve muscle oxygen delivery and contractile properties. In addition, beetroot supplementation increased circulating nitrate and nitrite concentrations, supporting the physiological relevance of the intervention. Collectively, these findings suggest potential clinical relevance for preventing age-related structural and neuromuscular functional decline in postmenopausal women.

## Figures and Tables

**Table 1:** Baseline characteristics of the participants.

	PLA (n = 10)	BET (n = 10)	*p*-Value *
Age (years)	67 ± 5	65 ± 5	0.941
Weight (kg)	67.6 ± 11.2	68.2 ± 12.3	0.933
Height (m)	1.61 ± 0.1	1.60 ± 0.1	0.976
BMI (kg/m^2^)	26.1 ± 3.7	26.7 ± 4.4	0.901
%BF	38.1 ± 9.9	35.6 ± 9.1	0.871
%MM	25.7 ± 4.5	26.1 ± 5.1	0.842
Peak torque (Nm·kg^−1^)	2.03 ± 0.65	2.29 ± 0.55	0.717
PAL (min/week)	221 ± 116	242 ± 109	0.728

Values are expressed as mean ± SD. %BF = percentage body fat; BMI = body mass index; %MM = percentage muscle mass; PAL = physical activity level. * An independent *t*-test was used to identify differences between BET and PLA groups.

## Data Availability

The original contributions presented in this study are included in the article. Further inquiries can be directed to the corresponding author.

## Supplementary Materials

The following supporting information can be downloaded at https://www.mdpi.com/article/10.3390/nu18050860/s1, Figure S1: Flow diagram illustrating the processes of recruitment, randomization, and follow-up throughout the randomized parallel trial; Table S1: Changes in muscle strength, muscle thickness, muscle quality, and rate of force development in the beetroot extract (BET) and placebo (PLA) groups at baseline (Pre), after 8 weeks, and after 12 weeks of supplementation; Table S2: Changes in serum nitrate and nitrite levels in the beetroot extract (BET) and placebo (PLA) groups at baseline (Pre), after 8 weeks, and after 12 weeks of supplementation; Table S3: Total daily nitrate and nitrite intake assessed by 24-h dietary recall in the beetroot extract (BET) and placebo (PLA) groups at baseline (Pre), after 8 weeks, and after 12 weeks of supplementation.
