# Supplementary material for: Long-Term Beetroot Extract Supplementation Improves Morphological Muscle Quality and Rate of Force Development in Postmenopausal Women: A Randomized Clinical Trial
Source: Nutrients. 2026 Mar 7;18(5):860. doi: 10.3390/nu18050860 (PMC12986568; doi:10.3390/nu18050860)
Supplement: Supplementary file 1 [file nutrients-18-00860-s001.zip › nutrients-4114023-supplementary.pdf]

nutrients

MDPI

Figure S1. Flow diagram illustrating the processes of recruitment, randomization, and follow-up throughout the randomized parallel trial.

Figure S1.

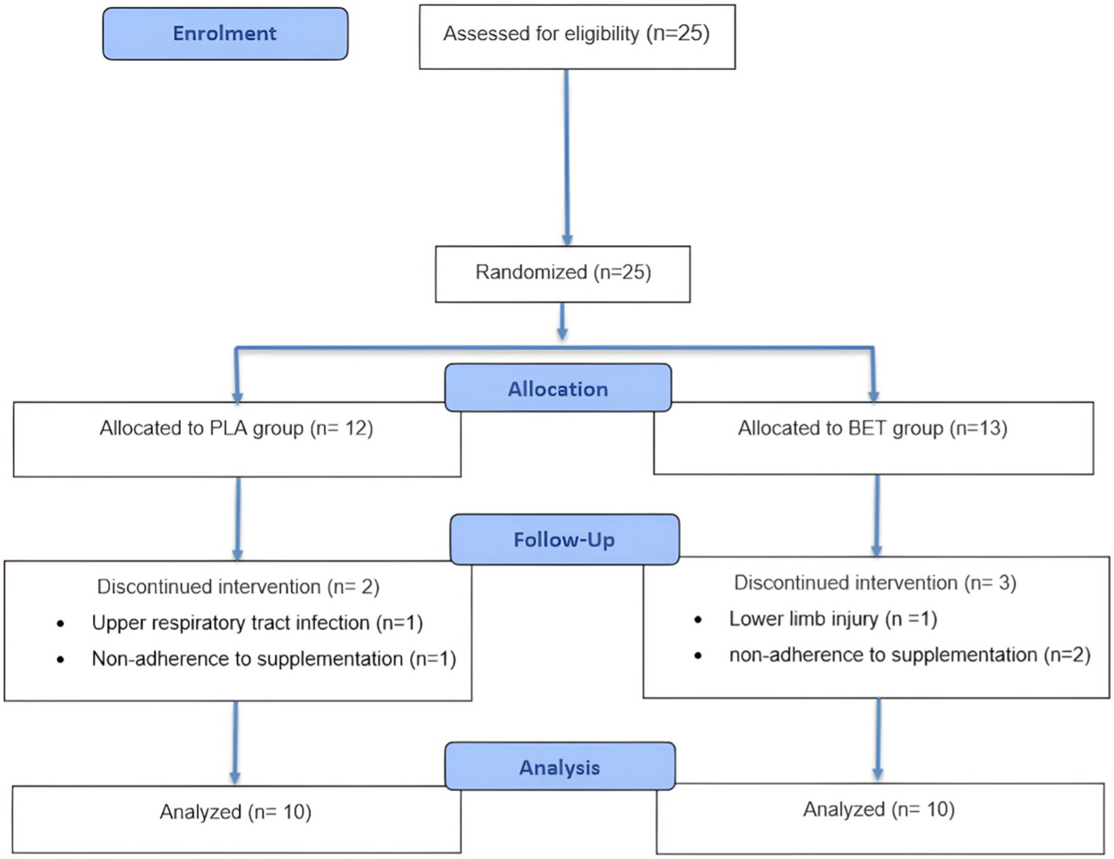

Table S1. Changes in muscle strength, muscle thickness, muscle quality, and rate of force development in the beetroot extract (BET) and placebo (PLA) groups at baseline (Pre), after 8 weeks, and after 12 weeks of supplementation.

**Variable**	**Group**	**Pre**	**8 weeks**	**12 weeks**	***P-value (interaction)***
MVIC (% change)	**BET**	100 ± 0	108.4 ± 9.5	120.3 ± 24.5[a]	0.189
	**PLA**	100 ± 0	106.4 ± 14.6	104.2 ± 17.6	
Muscle Thickness (cm)	**BET**	7.21 ± 1.1	7.97 ± 1.2[a]	8.03 ± 1.0[a]	0.145
	**PLA**	7.47 ± 0.4	7.87 ± 0.7	7.83 ± 1.0	
MQ_f (Nm.cm)	**BET**	10.7 ± 2.3	11.5 ± 1.9	12.2 ± 2.2[a]	0.436
	**PLA**	10.5 ± 2.1	11.0 ± 2.6	11.33 ± 2.21	
MQ_m (a.u.)	**BET**	146.8 ± 36.2	131.5 ± 30.7[a]	131.9 ± 31.3[a,*]	0.014

 https://doi.org/10.3390/nu1805086

	**PLA**	145.8 ± 20.5	145.3 ± 26.8	142.8 ± 25.0	
RFD_{30} (Nm.s¹)	**BET**	3.07 ± 1.1	5.91 ± 2.2[a,*]	6.29 ± 2.4[a,*]	< 0.001
	**PLA**	2.76 ± 1.7	2.56 ± 1.7	2.67 ± 1.9	
RFD_{50} (Nm.s¹)	**BET**	3.22 ± 1.6	5.39 ± 2.2 [a,*]	6.21 ± 2.0[a,*]	< 0.001
	**PLA**	3.1 ± 1.6	2.80 ± 1.9	2.85 ± 1.8	
RFD_{100} (Nm.s¹)	**BET**	3.02 ± 1.0	4.75 ± 1.9[a]	4.94 ± 1.5[a,*]	0.013
	**PLA**	2.59 ± 1.5	3.1 ± 1.9	3.28 ± 1.8	
RFD_{200} (Nm.s¹)	**BET**	3.26 ± 1.2	4.45 ± 1.5[a]	4.64 ± 1.4[a]	0.170
	**PLA**	2.66 ± 1.2	3.13 ± 1.8	3.42 ± 1.5	
RFD_{peak} (Nm.s¹)	**BET**	3.15 ± 0.9	4.78 ± 1.8[a,*]	5.00 ± 1.8[a,*]	0.015
	**PLA**	2.69 ± 1.3	3.13 ± 1.8	3.42 ± 1.5	

Data are mean ± standard deviation. MVIC = Maximal voluntary isometric contraction; MQ_f = functional muscle quality; MQ_m = morphological muscle quality; RFD = rate of force development. The symbol * denotes a significant difference (P<0.05) between groups. Different letters indicate statistical significance: [a] p<0.05 vs Pre. A repeated measures two-way ANOVA was used to identify differences between BET and PLA groups.

Table S2. Changes in serum nitrate and nitrite levels in the beetroot extract (BET) and placebo (PLA) groups at baseline (Pre), after 8 weeks, and after 12 weeks of supplementation.

**Variable**	**Group**	**Pre**	**8 weeks**	**12 weeks**	***P-value (interaction)***
Nitrate (µmol.L^{-1})	**BET**	40.9 ± 31.4	251.0 ± 239.3[a,*]	219.0 ± 205.2[a,*]	0.005
	**PLA**	28.1 ± 10.4	36.13 ± 12.9	25.8 ± 9.1	
Nitrite (µmol.L^{-1})	**BET**	0.47 ± 0.22	0.82 ± 0.33[a,*]	0.67 ± 0.29[a,*]	0.022
	**PLA**	0.42 ± 0.24	0.42 ± 0.29	0.47 ± 0.24	

Data are mean ± standard deviation. The symbol * denotes a significant difference (P<0.05) between groups. Different letters indicate statistical significance: [a] p<0.05 vs Pre. A repeated measures two-way ANOVA was used to identify differences between BET and PLA groups.

Table S3. Total daily nitrate and nitrite intake assessed by 24-h dietary recall in the beetroot extract (BET) and placebo (PLA) groups at baseline (Pre), after 8 weeks, and after 12 weeks of supplementation.

**Variable**	**Group**	**Pre**	**8 weeks**	**12 weeks**	***P-value (interaction)***

Dietary nitrate (mg/day)	**BET**	75 ± 20	33 ± 9[a]	26 ± 8[a]	0.412
	**PLA**	72 ± 18	35 ± 10[a]	28 ± 9[a]	
Nitrate from intervention (mg/day)	**BET**	—	548	548	—
	**PLA**	—	43	43	
Total nitrate intake (mg/day)	**BET**	75 ± 20	581 ±9 [a,*]	574 ± 8[a,*]	<0.001
	**PLA**	72 ± 18	78 ± 10	71 ± 9	

Data are mean ± standard deviation. The symbol * denotes a significant difference ($P<0.05$) between groups. Different letters indicate statistical significance: [a] $p<0.05$ vs Pre. A repeated measures two-way ANOVA was used to identify differences between BET and PLA groups.

https://doi.org/10.3390/nu1805086
